# Supplementary material for: Transcriptome Profiling of the Resistance Response of Musa acuminata subsp. burmannicoides, var. Calcutta 4 to Pseudocercospora musae
Source: Int J Mol Sci. 2022 Nov 5;23(21):13589. doi: 10.3390/ijms232113589 (PMC9657955; doi:10.3390/ijms232113589)
Supplement: Supplementary file 1 [file ijms-23-13589-s001.zip › SUPP_TABLE_S3_PRIMERS_RT-qPCR.pdf]

Supplementary Table S3. Selected target genes, primer sequences and supporting information for qRT-PCR validation of *in silico* differential gene expression.

**Target reference *Musa acuminata* DH Pahang Gene ID**	**Predicted gene function**	**Forward Primer (5'-3')**	**Reverse Primer (5'-3')**	**Expected Amplicon size (bp)**
Ma09_g20710	Chitinase 6	AAGAACCCAGCGACAGTCAA	AGTGAGGTTGTCTCCGGTGT	87
Ma01_g02630	Endochitinase	TGGAGACAACTTGGACTGCT	AAGCCGACACACATTTAGCC	87
Ma00_g04650	SNF1-Related Protein Kinase	CCCAATCGAAAATGGAATTG	GTCCAGTTGTCCCATGATCC	87
Ma06_g12740	Mitogen-activated protein kinase kinase 9	GACCCCTGCGACTCCTATGT	TAGTCCCCACCGTACGACTC	80
Ma07_g14030	WRKY Transcription factor 41	ACATGCACGATCAAGTCCTG	GAGAAGACACGGCTTTGAGG	85
Ma06_g35820	Laccase 24	CGCAACACAGTCCAAGACAT	CCCTCTATCGCCGTGTTCT	86
Ma01_g19550	Pathogenesis-related Protein 1	CACGCTGAGGTACAAGACGA	TCGCTCAACGCTTCATATCT	90
Ma03_g08140	Pathogenesis-related Protein 1	TCTCTCTCTCTCAGGCTTTCG	TACGACAACCAAACGCACTC	84
Ma11_g20360	Protein phosphatase 2C 68	AGGAAGGGATGTGTTGAAGG	TCGTACCCACATTGCCTGTA	87
Ma09_g10840	Dirigent Protein 11	CTCGTCTTCCTTGGCATCAT	AGAAATGCAGGTGGGTCATC	82
Ma09_g10850	Dirigent Protein 11	CTCCCTCTCTGCCTTTGTTG	AGAAGTGGAGGTGCGTCATC	80
Ma09_g10830	Dirigent Protein 21	CCATGGCGTTCTCCTCTCTA	ATCTGCAGCGCCAAAAGAG	83
Ma01_g21800	AP2/ERF and B3 domain-containing transcription repressor RAV2	GATGTGACGGAGGGTAATGG	TCCTCAACAGCTCCCTTGAT	85
Ma08_g22530	EID1-like F-Box 3	TGGTGCATTCTCGCTTGTTA	AATTGGGACTGATCGCATTC	87
